# PfMFR3: A Multidrug-Resistant Modulator in *Plasmodium falciparum*

**DOI:** 10.1021/acsinfecdis.0c00676

**Published:** 2021-03-15

**Authors:** Frances Rocamora, Purva Gupta, Eva S. Istvan, Madeline R. Luth, Emma F. Carpenter, Krittikorn Kümpornsin, Erika Sasaki, Jaeson Calla, Nimisha Mittal, Krypton Carolino, Edward Owen, Manuel Llinás, Sabine Ottilie, Daniel E. Goldberg, Marcus C. S. Lee, Elizabeth A. Winzeler

**Affiliations:** † Department of Pediatrics, School of Medicine, University of California, San Diego, La Jolla, California 92093, United States; ‡ VA San Diego Healthcare System, Medical and Research Sections, La Jolla, California 92161, United States; § Department of Medicine, Division of Pulmonary and Critical Care, University of California, San Diego, La Jolla, California 92037, United States; ∥ Departments of Medicine and Molecular Microbiology, Washington University School of Medicine, St. Louis, Missouri 63130, United States; ⊥ Wellcome Sanger Institute, Hinxton CB10 1SA, United Kingdom; # Department of Biochemistry and Molecular Biology, Pennsylvania State University, University Park, Pennsylvania 16802, United States; ¶ Huck Center for Malaria Research, Pennsylvania State University, University Park, Pennsylvania 16802, United States; ○ Department of Chemistry, Pennsylvania State University, University Park, Pennsylvania 16802, United States

**Keywords:** malaria, drug resistance, drug discovery, transporter, mitochondria

## Abstract

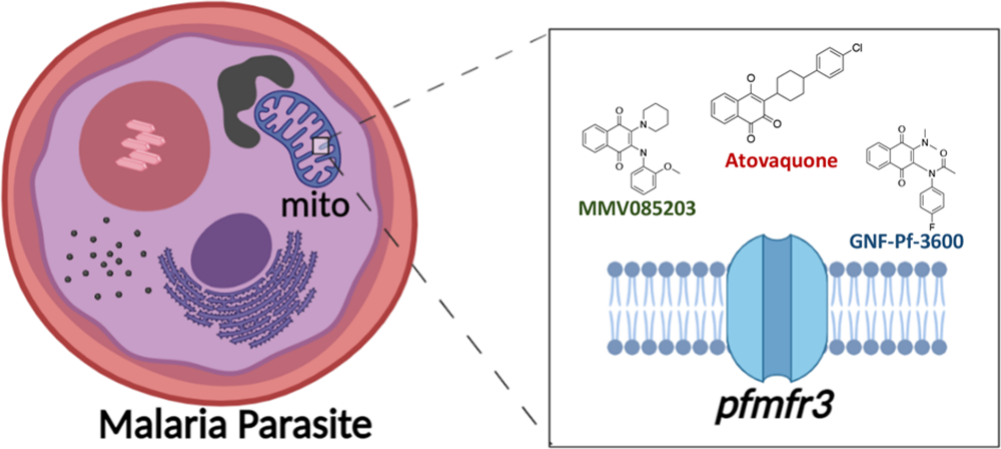

In malaria, chemical genetics is a powerful method for assigning function to uncharacterized genes. MMV085203 and GNF-Pf-3600 are two structurally related napthoquinone phenotypic screening hits that kill both blood- and sexual-stage *P. falciparum* parasites in the low nanomolar to low micromolar range. In order to understand their mechanism of action, parasites from two different genetic backgrounds were exposed to sublethal concentrations of MMV085203 and GNF-Pf-3600 until resistance emerged. Whole genome sequencing revealed all 17 resistant clones acquired nonsynonymous mutations in the gene encoding the orphan apicomplexan transporter PF3D7_0312500 (*pfmfr3*) predicted to encode a member of the major facilitator superfamily (MFS). Disruption of *pfmfr3* and testing against a panel of antimalarial compounds showed decreased sensitivity to MMV085203 and GNF-Pf-3600 as well as other compounds that have a mitochondrial mechanism of action. In contrast, mutations in *pfmfr3* provided no protection against compounds that act in the food vacuole or the cytosol. A dihydroorotate dehydrogenase rescue assay using transgenic parasite lines, however, indicated a different mechanism of action for both MMV085203 and GNF-Pf-3600 than the direct inhibition of cytochrome bc1. Green fluorescent protein (GFP) tagging of PfMFR3 revealed that it localizes to the parasite mitochondrion. Our data are consistent with PfMFR3 playing roles in mitochondrial transport as well as drug resistance for clinically relevant antimalarials that target the mitochondria. Furthermore, given that *pfmfr3* is naturally polymorphic, naturally occurring mutations may lead to differential sensitivity to clinically relevant compounds such as atovaquone.

With more than 200 million cases and over 400,000 deaths globally, malaria remains a devastating disease and gross burden on public health.^1^ It is caused by protozoan parasites belonging to the *Plasmodium* genus and is transmitted by female anophelene mosquitoes. Although substantial effort and resources have been mustered toward the aim of eradicating malaria, the inevitable emergence of drug resistance remains a significant obstacle to complete and lasting malaria control. Not only has the diminished efficacy of available therapeutics necessitated the discovery and development of new candidate antiplasmodial compounds, but also it has underscored the need for a better understanding of the biological correlates of drug resistance in malaria, dubbed the “malaria resistome”. As key components of the malaria resistome, transport proteins are often involved in drug response phenotypes^2−4^ either as the targets of the drug themselves^5,6^ or by helping the parasite evade drug action.^7^ Of the ∼120 members of the *P. falciparum* transportome, many remain unexplored, pending experimental characterization of their specific function and subcellular localization.^4,8^ To address this, the forward chemical genetic approach of inducing drug resistance *in vitro* has revealed a plethora of novel phenotypes associated with drug resistance, which can in turn provide insight into the physiological role of these transport proteins.^2,3,9^ In this technique, drug resistance is first selected through prolonged exposure to sublethal concentrations of a compound. Next, the genomes of the resistant clones are compared to that of their isogenic parents to discover genetic changes that are likely to be responsible for modulating a drug response.

With the help of *in vitro* evolution, we were able to identify, characterize, and validate a novel putative transporter as a key mediator of resistance against two structurally similar naphthoquinone derivatives, MMV085203^10^ (alternative name: GNF-Pf-4450) and GNF-Pf-3600,^11^ that were identified from previous phenotypic screens (Figure 1a). Not only are both MMV085203 and GNF-Pf-3600 active against the asexual blood stage of the parasite life cycle,^11,12^ which is responsible for the clinical manifestation of malaria, but also they inhibit the growth of the mature sexual form of the parasite (MMV085203 IC_50_ = 2.9 μM, GNF-Pf-3600 IC_50_ = 0.1 μM), which is subsequently transmitted by the mosquito vector across human hosts.^13,14^ Through cross-resistance profiling of evolved drug-resistant parasites, we were also able to identify the localization for this transporter and elucidate its potential as a facilitator of multidrug resistance in *P. falciparum*.

**Figure 1:**
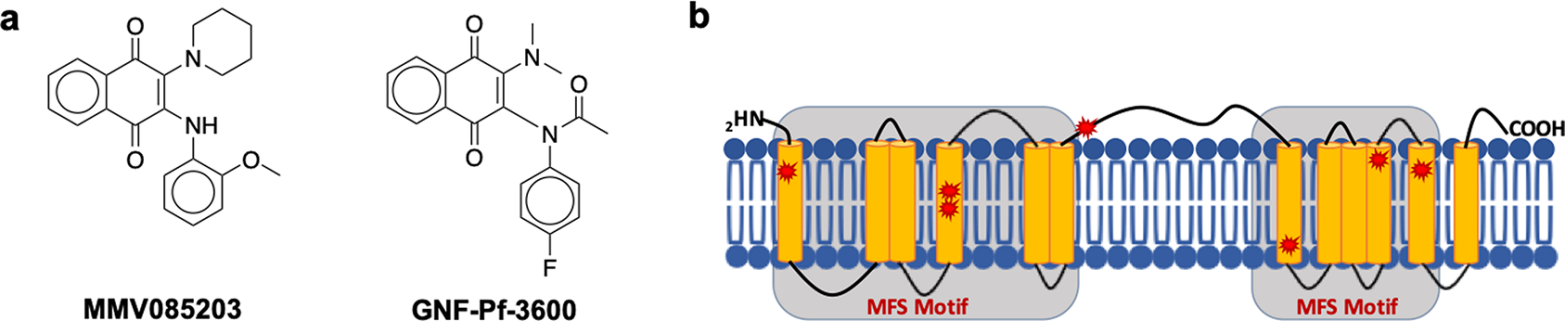
Mutations in *pfmfr3* are associated with resistance to MMV085203 and GNF-Pf-3600. (a) Chemical structures of MMV085203 and GNF-Pf-3600, which were used for *in vitro* selection against *Plasmodium falciparum*. (b) Protein schematic of PfMFR3 together with the mutations identified by *in vitro* evolution and whole genome analysis. Predicted transmembrane domains are marked in yellow, and mutations are marked in red stars.

## Results

### *In Vitro* Evolution of *P. falciparum* Resistance to MMV085203 and GNF-Pf-3600

To investigate the mechanism of action and resistance to these two structurally related molecules, we generated resistant parasite lines using *in vitro* evolution. Parasites from a 3D7 and Dd2 background of *P. falciparum* were subjected to intermittent treatment with increasing concentrations of GNF-Pf-3600 over the course of four months, resulting in 6 clones each from 3D7 and Dd2 that are up to 5- to 10-fold and 2- to 3-fold less sensitive to GNF-Pf-3600, respectively (Table 2). In a similar fashion, 3D7 strain parasites were also exposed to a stepwise dose progression of MMV085203 from a starting dose of 1 × IC_50_ up to 6 × IC_50_ over a period of 6 months. Drug selection yielded five clonal lines that had 4- to 6-fold higher IC_50_ values against MMV085203 compared to the parent (Table 1).

**Table 1:** 72 h IC_50_ Values of MMV085203-Resistant Clones Generated from *in Vitro* Evolution

MMV085203	IC_50_ (nM)	fold shift
3D7 parent	54.9 ± 27	
3D7-1F2	252.0 ± 114.3	5
3D7-1G5	311.6 ± 108.3	6
3D7-2B3	289.6 ± 86.1	5
3D7-3B3	213.9 ± 85.4	4
3D7-3F3	241.8 ± 78.1	4

**Table 2:** 72 h IC_50_ Values of GNF-Pf-3600-Resistant Clones Generated from *in Vitro* Evolution

	IC_50_ (nM)	fold shift
3D7 parent	20.0 ± 1. 5	
3D7-3B8	196.9 ± 7.0	10
3D7-3E3	105.5 ± 4.0	5
3D7-3H7	158.8 ± 10.4	8
3D7-4A5	147.0 ± 21.9	7
3D7-4A8	103.3 ± 3.0	5
3D7–4G6	166.4 ± 7.8	8
		
Dd2 parent	34.8 ± 2.2	
Dd2-3D2	45.3 ± 5.1	1.3
Dd2-4A8	79.7 ± 13.1	2
Dd2-4B10	91.9 ± 8.8	3
Dd2-4F4	59.1 ± 3.7	2
Dd2-3H5	74.0 ± 1.2	2
Dd2-3C11	79.9 ± 2.7	2

To identify possible targets or markers of resistance for the compounds, all 17 resistant clones were subjected to whole genome sequencing to 40–100× coverage. After applying stringent filtering (see the Methods), we detected 13 indels and 21 single nucleotide variants of which 30 were nonsynonymous mutations. Looking only at intragenic mutations that were detected after stringent filtering and not found in the nonselected parental lines, we found 12 variants in 9 genes among the MMV085203-resistant clones. The 12 independent GNF-Pf-3600-resistant clones derived from 3D7 and Dd2 bore nonsynonymous mutations in 15 and 5 genes, respectively. Strikingly, all clonal lines that acquired resistance to either compound contained nonsynonymous mutations in PF3D7_0312500 (*pfmfr3*), a gene predicted to encode an uncharacterized, putative transporter MFR3 (Tables 3 and 4). Mutations in *pfmfr3* were found among all clones that acquired resistance to MMV085203 and GNF-Pf-3600, which demonstrates a significant enrichment of genetic changes in this gene compared to other genes in our data set (*p* < 2 × 10^–16^). Additionally, a single nonsynonymous alteration in this gene was sufficient to confer resistance to MMV085203 in clone 3D7-2B3 (Q487E) and GNF-Pf-3600 in clone Dd2-4B10 (D150V). It is noteworthy that mutations in *pfmfr3* have not been identified with prior selections using other compounds.^3,9,15−19^ We also searched for copy number variations (CNVs) by normalizing the mean coverage of coding regions for each clone against their corresponding parental background (3D7 or Dd2) and identifying sections of the genome having two or more contiguous genes that had at least a 2-fold difference in read depth against the parent. On the basis of these parameters, however, we did not detect CNVs in this particular data set.

**Table 3:** SNVs Obtained from the *in Vitro* Evolution of a 3D7 Strain of *P. falciparum* against MMV085203

gene name	description	effect	3D7-1F2	3D7-1G5	3D7-2B3	3D7-3B3	3D7-3F3
PF3D7_0221700	Plasmodium exported protein, unknown function	D63_G64insPKPSTLNP		x		x	
PF3D7_0312500	major facilitator superfamily related transporter, putative	C401Y	x				x
PF3D7_0312500	major facilitator superfamily related transporter, putative	Q487E			x		
PF3D7_0312500	major facilitator superfamily related transporter, putative	S519stop		x			
PF3D7_0312500	major facilitator superfamily related transporter, putative	N279frameshift				x	
PF3D7_0718000	dynein heavy chain, putative	intronic indel				x	
PF3D7_0812100	conserved protein, unknown function	T1326_T1335del	x				x
PF3D7_0823000	serine/threonine protein kinase VPS15, putative	N830K		x		x	
PF3D7_0918800	dihydrouridine synthase, putative	N526D	x				x
PF3D7_1227200	potassium channel	D1330Y		x			
PF3D7_1233600	asparagine- and aspartate-rich protein 1	D3256_N3262del				x	
PF3D7_1372200	histidine-rich protein III	H119_H124del	x				x

**Table 4:** SNVs Obtained from the *in Vitro* Evolution of 3D7 and Dd2 Strains of *P. falciparum* against GNF-Pf-3600

gene name	description	effect	3D7-3B8	3D7-3E3	3D7-3H7	3D7-4A5	3D7-4A8	3D7-4G6
PF3D7_0305500	conserved Plasmodium protein, unknown function	D1228_D1229del	x	x		x	x	x
PF3D7_0307900	conserved Plasmodium protein, unknown function	L2968F	x	x	x	x	x	x
PF3D7_0307900	conserved Plasmodium protein, unknown function	D1648_Q1650dup	x	x	x	x	x	x
PF3D7_0312500	major facilitator superfamily related transporter, putative	G146R	x	x	x	x	x	x
PF3D7_0501400	interspersed repeat antigen	Q127Q				x		
PF3D7_0614300	organic anion transporter	intron variant	x	x		x		x
PF3D7_0929000	conserved Plasmodium protein, unknown function	intron variant				x		
PF3D7_1106600	DEAD/DEAH box helicase, putative	N90_N91dup				x		
PF3D7_1132400	conserved Plasmodium membrane protein, unknown function	D1030_N1031del	x	x	x	x	x	x
PF3D7_1118500	box C/D snoRNP rRNA 2′-O-methylation factor, putative	H545Y				x	x	
PF3D7_1222600	transcription factor with AP2 domain(s)	S2162R						x
PF3D7_1222800	conserved Plasmodium protein, unknown function	intron variant	x	x	x	x	x	x
PF3D7_1314300	conserved Plasmodium protein, unknown function	S197S	x	x	x	x	x	x
PF3D7_1363400	polyubiquitin binding protein, putative	N220_N221dup					x	x
PF3D7_1408200	transcription factor with AP2 domain(s)	N900_N901del	x	x	x	x	x	
PF3D7_1470100	conserved Plasmodium protein, unknown function	L2246S				x		x

gene name	description	effect	Dd2-3C11	Dd2-3D2	Dd2-3H5	Dd2-4A8	Dd2-4B10	Dd2-4F4
PF3D7_0310200	phd finger protein, putative	V2606frameshift	x	x	x	x		x
PF3D7_0312500	major facilitator superfamily related transporter, putative	S16R	x	x	x			
PF3D7_0312500	major facilitator superfamily related transporter, putative	D150V				x	x	x
PF3D7_0402300	reticulocyte binding protein homologue 1	K1244N			x			
PF3D7_0515400	conserved Plasmodium protein, unknown function	C174S	x	x	x			
PF3D7_1254500	rifin|PIR protein	Q4H		x				x

An orphan, previously uncharacterized transporter, MFR3, bears very little overall sequence similarity to any other known protein in current databases. On the basis of its general topology, this 579-amino acid protein is classified under the major facilitator superfamily (MFS) of transporters^20^ due to the presence of an MFS-like motif, which is characterized by 12 transmembrane helices divided into 2 distinct domains on the N and C terminal ends of the protein, with each domain consisting of 6 consecutive transmembrane segments^20−22^ (Figure 1b). Six out of the 7 nonsynonymous variants identified among the clones are located in the predicted transmembrane regions of the protein (S16R, G146R, D150V, C401Y, Q487E, and S519stop), while one frameshift mutation (N279 fs) is found right after the sixth transmembrane segment. All of these findings indicate that this novel parasite MFS transporter is crucial to regulating parasite sensitivity against MMV085203 and GNF-Pf-3600.

### *pfmfr3* Mediates Resistance to MMV085203, GNF-Pf-3600, and Atovaquone

**Figure 2:**
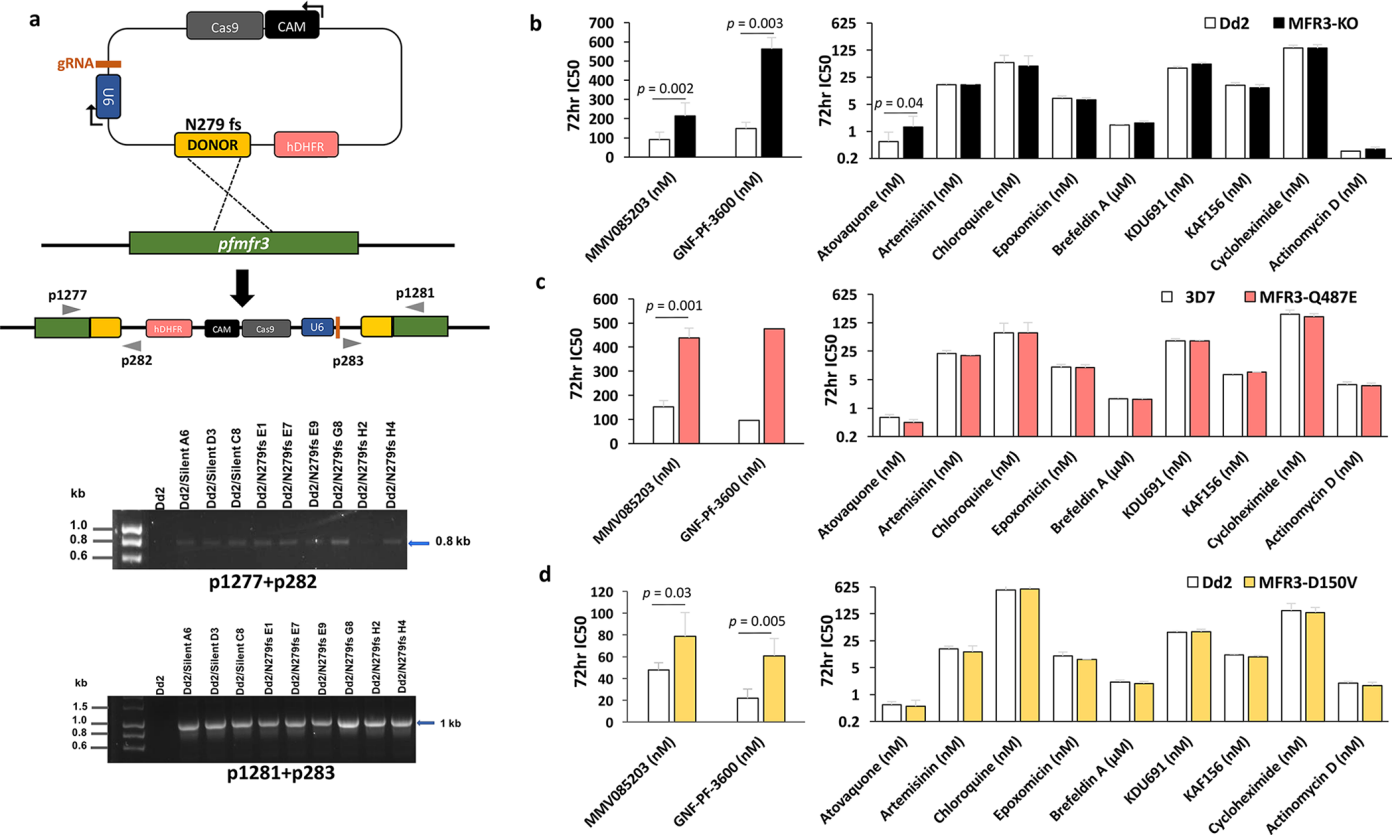
Disruption of *pfmfr3* confers resistance to MMV085203 and GNF-Pf-3600. (a) Map of plasmid used for CRISPR-Cas9 editing of endogenous *pfmfr3* accompanied by the evidence of complete donor plasmid recombination into the genomic *pfmfr3* locus. Integration on the 5′ and 3′ ends of the gene is demonstrated by the p1277+p282 and p1281+p283 amplicons, respectively. Dd2/N279 fs represents clones transfected with the donor containing the mutation, while Dd2/silent represents clones transfected with a silent control donor plasmid. (b) The sensitivity of the CRISPR-edited *pfmfr3* mutant expressing the truncated form of the protein (MFR3-KO) was evaluated against MMV085203, GNF-Pf-3600, and other antimalarial compounds with known mechanisms of action with wild-type Dd2 (Dd2) as a control; additional data can be found in Table 5. The sensitivity of the evolved (c) MMV085203-resistant mutant (*pfmfr3* Q487E) and the (d) GNF-Pf-3600-resistant mutant (*pfmfr3* D150V) was also evaluated against the same set of compounds as MFR3-KO, in comparison to their respective wild-type 3D7 and Dd2 parent lines; additional data can be found in Table 6. Bars represent the mean ± SD IC_50_ values from at least three independent biological replicates. Pairwise comparisons between parasite lines were performed using the Student’s *t* test.

To further validate the link between *pfmfr3* and the resistance phenotype we observed in the *in vitro*-evolved parasite lines, we introduced the N279 frameshift mutation into a wild-type Dd2 background using CRISPR-Cas9 (Figure 2a). Originally identified in a clone (3D7-3B3) that was 4-fold more resistant to MMV085203, this frameshift event results in a N279I change as well as a premature stop codon at position 281, effectively truncating the gene product from 579 to 280 amino acids, resulting in the loss of the last six transmembrane domains and likely disrupting its function. Although we were able to confirm editing in all clones transfected with both the N279 fs plasmid and the silent control plasmid, all attempts at PCR amplification of the entire gene were not successful. Alternatively, PCR genotyping was performed in order to detect the possible recombination of the entire plasmid into the parasite genome, resulting from CRISPR-induced double-strand break and homologous-directed repair. We found that all transgenic clones underwent disruption of endogenous *pfmfr3* by plasmid integration into the genome, which is detected by p1277+p282 and p283+p1281 primer pairs (Figure 2a). Nevertheless, Sanger sequencing of the targeted segment of *pfmfr3* revealed that the intact donor sequence was integrated into the gene as expected, thereby still resulting in a premature stop codon at position 281 effectively shortening the gene product and presumably leading to the loss of protein function.

Comparing the drug sensitivity of the edited *pfmfr3* mutant line against wild-type Dd2, we observed that the clone expressing the truncated protein was 2.5- and 4-fold less sensitive to both MMV085203 (*p* = 0.002) and GNF-Pf-3600 (*p* = 0.003), respectively (Figure 2b, Table 5), demonstrating the role of this putative transporter as an important modulator of drug response to both compounds. Additionally, we found clone 3D7-2B3, which was specifically evolved against MMV085203 and bore a single Q487E mutation in *pfmfr3*, to also be cross-resistant against GNF-Pf-3600 (Figure 2c, Table 6). Likewise, resistant clone Dd2-4B10, which was specifically exposed to GNF-Pf-3600 and contained a single D150V mutation in *pfmfr3*, was also significantly less sensitive to MMV085203 (Figure 2d, Table 6).

**Table 5:** 72 h IC_50_ Values of Dd2 vs MFR3-KO Strains against Antimalarial Compounds

IC_50_	Dd2	MFR3-KO
MMV085203 (nM)	90.0 ± 39.7	213.9 ± 68.6
GNF-Pf-3600 (nM)	148.5 ± 32.5	563.5 ± 58.6
atovaquone (nM)	0.550 ± 0.4	1.31 ± 1.10
artemisinin (nM)	15.9 ± 1.2	16.2 ± 0.5
chloroquine (nM)	59.4 ± 32.5	47.7 ± 41.6
epoxomicin (nM)	7.19 ± 1.00	6.55 ± 0.8
brefeldin A (μM)	1.48 ± 0.00	1.66 ± 0.2
KDU691 (nM)	43.2 ± 3.80	54.4 ± 5.4
KAF156 (nM)	15.7 ± 2.30	13.3 ± 2.8
cycloheximide (nM)	143.9 ± 24.0	140.2 ± 32.7
actinomycin D (nM)	0.310 ± 0.10	0.340 ± 0.00

**Table 6:** 72 h IC5_50_ Values of 3D7 vs MFR3-Q487E (3D7 Background) and Dd2 vs MFR3-D150V (Dd2 Background) against Antimalarial Compounds

IC_50_	3D7	MFR3-Q487E	Dd2	MFR3-D150V
MMV085203 (nM)	152.7 ± 25.8	438.4 ± 40.8	47.6 ± 6.8	78.6 ± 21.9
GNF-Pf-3600 (nM)^a^	96.1	476.6	21.7 ± 8.6	60.8 ± 15.9
atovaquone (nM)	0.585 ± 0.11	±0.09	0.546 ± 0.11	0.497 ± 0.21
artemisinin (nM)	21.7 ± 4.3	19.5 ± 0.95	15.4 ± 2.8	12.9 ± 5.6
chloroquine (nM)	70.2 ± 51.2	71.4 ± 54.9	556.8 ± 197.6	556.1 ± 224.2
epoxomicin (nM)	10.1 ± 1.8	9.82 ± 1.8	10.1 ± 2.6	8.17 ± 0.15
brefeldin A (μM)	1.71 ± 0.09	1.63 ± 0.11	2.13 ± 0.25	1.94 ± 0.26
KDU691 (nM)	45.0 ± 5.74	44.4 ± 2.19	41.7 ± 3.0	42.8 ± 7.2
KAF156 (nM)	6.68 ± 0.38	7.60 ± 0.10	10.7 ± 0.51	9.6 ± 0.73
cycloheximide (nM)	202.9 ± 53.2	174.4 ± 34.3	152.2 ± 80.3	135.3 ± 47.5
actinomycin D (nM)	3.74 ± 0.67	3.58 ± 0.55	1.98 ± 0.2	1.73 ± 0.3

a Only one biological replicate.

We also sought to gain some insight into the parasiticidal mechanism of these two structurally related compounds by assaying the sensitivity of *pfmfr3*-mutated clones against nine compounds that have antimalarial activity and exhibit different modes of drug action. In addition to the functional knockout line, which bears the nonsense mutation, we also tested the evolved clones 3D7-2B3 and Dd2-4B10 against the same panel of antimalarial drugs. The truncated-MFR3 clone showed differential decreased sensitivity to atovaquone (*p* = 0.04) compared to wild-type Dd2, while remaining similarly sensitive to artemisinin, chloroquine, epoxomicin, brefeldin A, KDU691, KAF156, cycloheximide, and actinomycin D (Figure 2a, Table 5). On the other hand, no other cross-resistance phenotypes were observed in the case of the Q487E and D150V mutants (Figure 2c,d, Table 6). The significant decrease in sensitivity to atovaquone, which also contains a 1,4-naphthoquinone scaffold like MMV085203 and GNF-Pf-3600, observed in the functional knockout line suggests a possible role for MFR3 in the transport of this frontline antimalarial drug. On the other hand, the lack of resistance observed in the Q487E and D150V point mutants against atovaquone suggests differences in which specific transmembrane domains are responsible for the binding and transport among these three compounds.

### MMV085203 and GNF-Pf-3600 Do Not Inhibit Cytochrome bc1

**Figure 3:**
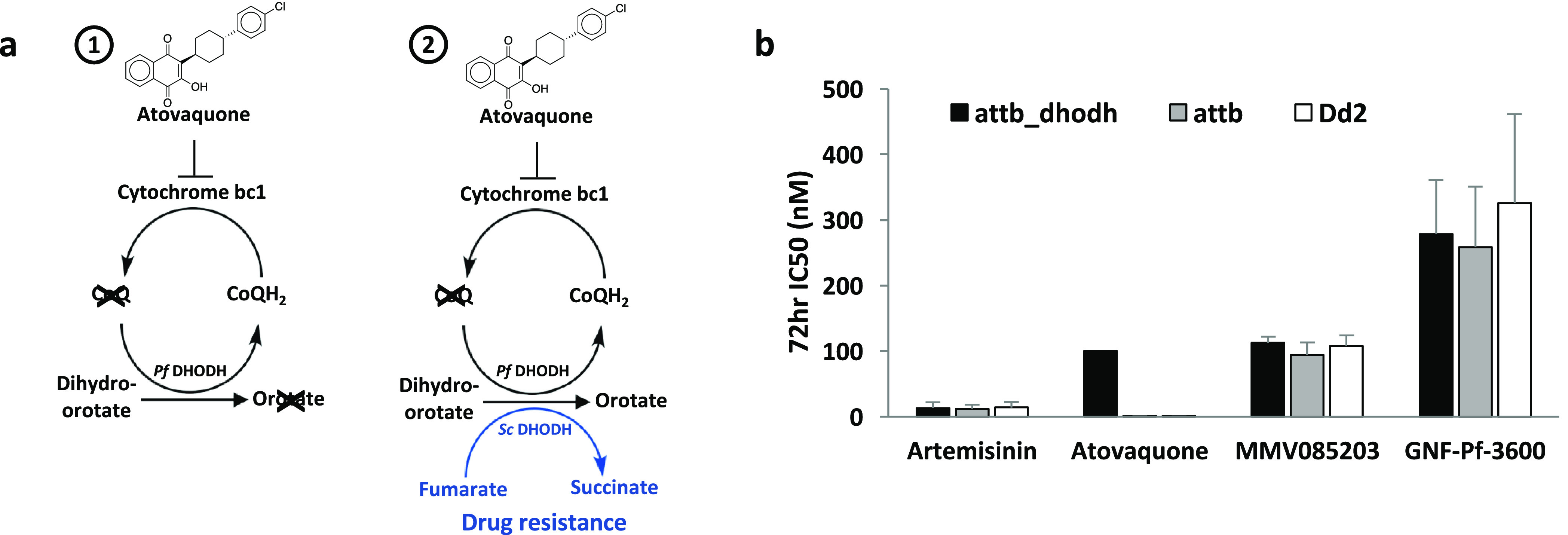
MMV085203 and GNF-Pf-3600 do not target the mitochondrial electron transport chain. (a) Simplified schematic of the mitochondrial electron transport chain (mETC) and pyrimidine synthesis in *P. falciparum* in the (1) absence and (2) presence of *Sc*DHODH. The loss of ubiquinone (CoQ) due to the inhibition of cytochrome bc1 by atovaquone results in the downstream obstruction of the CoQ-mediated conversion of dihydroorotate to orotate by *Pf*DHODH. However, genetic supplementation of CoQ-independent *Sc*DHODH is able to bypass this blockage, rendering the parasite resistant against cytochrome bc1 inhibition. (b) The sensitivity against artemisinin (noncytochrome bc1 inhibitor), atovaquone (cytochrome bc1 inhibitor), MMV085203, and GNF-Pf-3600 was measured across three parasite lines: the transgenic *P. falciparum* line overexpressing yeast DHODH generated using a Dd2 line bearing an attb recombination site (attb_dhodh), *P. falciparum* bearing an *attB* recombination site on a Dd2 background (attb), and a wild-type Dd2 strain (Dd2). Additional data can be found in Table 7. Bars represent mean ± SD IC_50_ values from three independent biological replicates.

On the basis of the shared structural features across atovaquone, MMV085203, and GNF-Pf-3600 as well as the alteration in sensitivity for all three compounds resulting from the disruption of *pfmfr3*, we explored the possibility of MMV085203 and GNF-Pf-3600 having the same mechanism of action as atovaquone, a clinically relevant antiparasitic drug that targets the cytochrome bc1 complex in *Plasmodium* parasites through competitive inhibition of ubiquinol.^23,24^ Not only does blockage of cytochrome bc1 by atovaquone disrupt the mitochondrial electron transport chain (mETC), but also it triggers the downstream inhibition of pyrimidine biosynthesis due to the loss in production of ubiquinone (CoQ), a molecule that is regenerated through the mETC and is in turn the substrate of *Pf* dihydroorotate dehydrogenase (DHODH), the enzyme responsible for the conversion of dihydroorotate to orotate, a pyrimidine precursor^25^ (Figure 3a). The parasiticidal action of atovaquone, therefore, is driven by the inhibition of these essential biological processes.

To investigate, we conducted a genetic supplementation assay that utilizes a transgenic parasite line that overexpresses the *S. cerevisiae*-derived DHODH enzyme (attb_dhodh).^25^ Unlike the parasite DHODH (*Pf*DHODH), the yeast-derived DHODH (*Sc*DHODH) is able to catalyze orotate production even in the absence of ubiquinone, effectively bypassing the mETC.^25,26^ Parasites that also express yeast DHODH, in addition to their own, are therefore refractory to compounds that act by disrupting the mETC through cytochrome bc1 inhibition, such as atovaquone (Figure 3a). Accordingly, we compared the 72 h IC_50_ for MMV085203 and GNF-Pf-3600 from a parasite line expressing ScDHODH (attb_dhodh) against the Dd2_attb parent (attb) from which it was derived, which only expresses the parasite version of the enzyme (*Pf*DHODH). Both strains are derived from a wild-type Dd2 background and contain an *attB* site designed for chromosomal integration.^27^ We also included a wild-type Dd2 as a control to rule out any possible phenotypic interference that could be due to the presence of the *attB* sequence. As expected, genetic supplementation of ScDHODH in the attb_dhodh parasites rendered them over 100-fold resistant against atovaquone relative to the attb and wild-type Dd2 parasites. In contrast, IC_50_ values for MMV085203 and GNF-Pf-3600 were comparable between the three parasite lines. The same outcome was likewise observed in the case of artemisinin, a potent antimalarial that does not target cytochrome bc1^28−30^ (Figure 3b, Table 7). This finding demonstrates that, despite sharing a 1,4-naphthoquinone scaffold with atovaquone, the mechanism of action of MMV085203 and GNF-Pf-3600 does not involve the specific inhibition of cytochrome bc1.

**Table 7:** 72 h IC_50_ Values of Dd2-attb_dhodh, Dd2-attb, and Dd2 Strains

IC_50_ (nM)	Dd2-attb_dhodh	Dd2-attb	Dd2
artemisinin	13.3 ± 8.7	12.1 ± 6.3	13.8 ± 8.7
atovaquone	>100^a^	0.27 ± 0.1	0.25 ± 0.1
MMV085203	112.3 ± 9.7	94.6 ± 18.7	107.9 ± 16.1
GNF-Pf-3600	278.8 ± 82.3	258.6 ± 92.3	326 ± 135.4

a Incomplete curve fitting. 50% inhibition observed at >100 nM.

### PfMFR3 Is a Putative Mitochondrial Transporter

**Figure 4:**
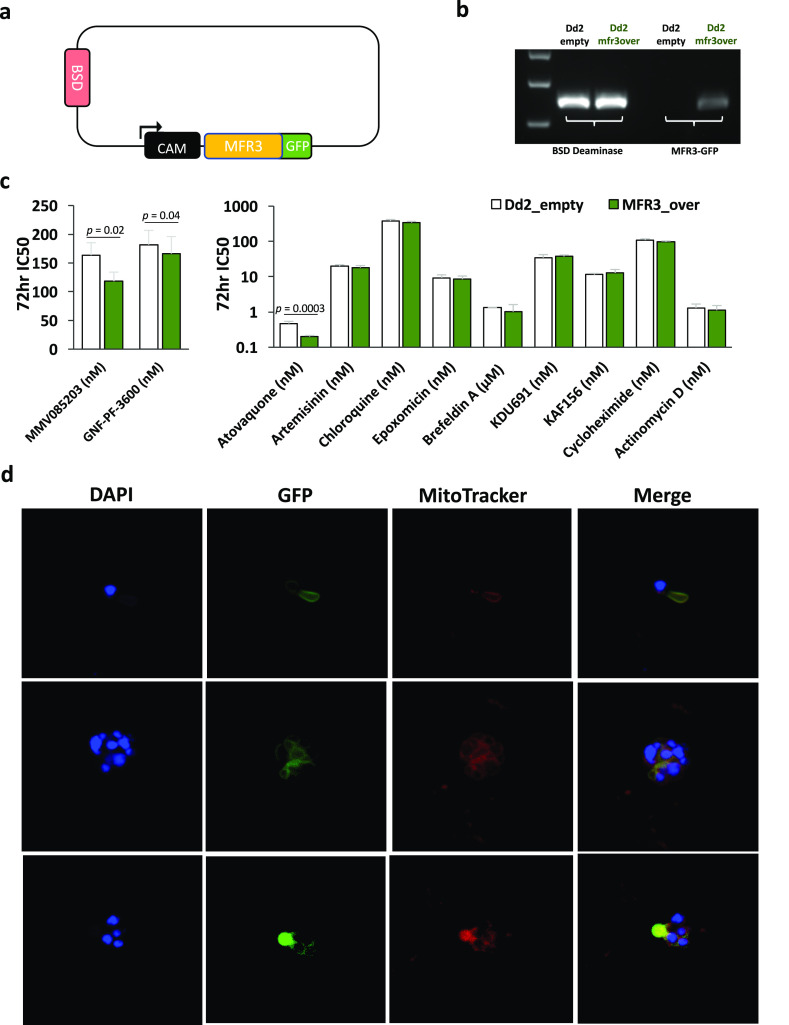
PfMF3 localizes to the parasite mitochondrion. (a) Map of plasmid used for episomal overexpression of GFP-tagged *pfmfr3*. An empty vector (no *pfmfr3* insert) was used for transfection of a control (Dd2_empty) parasite line. (b) PCR amplification of the blasticidin-resistance (BSD Deaminase) marker in both parasite lines confirms the successful transfection of both control and overexpression/tagging parasite lines, while PCR amplification of the chimeric MFR3-GFP template confirms the presence of the MFR3-GFP episome in the overexpression line (Dd2_mfr3over) but not in the control (Dd2_empty). (c) The sensitivity of parasites overexpressing *pfmfr3* (Dd2-MFR3_over) and the corresponding control line (Dd2_empty) was evaluated against MMV085203, GNF-Pf-3600, and other antimalarial compounds with known mechanisms of action. Bars represent mean ± SD IC_50_ values from at least three independent biological replicates. Pairwise comparisons between parasite lines were performed using the Student’s *t* test. Additional data can be found in Table 9. (d) Blood-stage parasites expressing a GFP-tagged version of PfMFR3 were costained with MitoTracker Red (200 nM) and DAPI and then imaged using confocal microscopy. The blue signal pertains to the DAPI-stained parasite nuclei; the red signal represents the parasite mitochondrion, and the green signal, GFP-tagged PfMFR3.

Finally, we sought to determine the cellular localization of MFR3 through the episomal overexpression of a green fluorescent protein (GFP)-tagged species of MFR3. The full-length coding sequence of this gene was generated through gene-specific PCR amplification of *pfmfr3* from total cDNA derived from a wild-type Dd2 clone and then inserted into a pDC2-*cam*-*mrfp*-2A–*gfp*^31^ vector backbone from which the mRFP-2A segment had been removed upstream of the GFP tag. Transfection of wild-type Dd2 with this construct leads to a parasite line that expresses MFR3 bearing a C-terminal GFP tag under the control of a constitutive calmodulin (CAM) promoter (Dd2_mfr3over) (Figure 4a). As a control, we also transfected a Dd2 parent with the empty vector, which was grown alongside the tagged MFR3 overexpression line (Dd2_empty). PCR genotyping of total DNA extracted from the resulting transfectants confirms the presence of the correct episome in both the empty vector and MFR3-GFP lines (Figure 4b), while quantitative PCR shows 3-fold overexpression of *pfmfr3* in the parasites transfected with the MFR3-GFP plasmid compared to the control line bearing only the empty vector (Table 8). Crucially, we were also able to observe a slight but reproducible increase in susceptibility against MMV05203 (*p* = 0.02) and GNF-Pf-3600 (*p* = 0.04) accompanying overexpression of MFR3 (Figure 4c, Table 9).

**Table 8:** Checking for *pfmfr3* Overexpression Using Quantitative PCR

	Dd2_MFR3over	Dd2_empty
endogenous + episomal *pfmfr3* (Ct)	22 ± 0.14	24.9 ± 0.34
arginyl tRNA synthetase (control) (Ct)	18.4 ± 0.02	19.8 ± 0.02
fold change *pfmfr3*	2.96 ± 0.89	

**Table 9:** 72 h IC_50_ Values of Dd2 vs MFR3_over

IC_50_	Dd2_empty	MFR3_over
MMV085203 (nM)	163.5 ± 21.9	118.2 ± 15.9
GNF-Pf-3600 (nM)	181.8 ± 24.9	166.2 ± 29.9
atovaquone (nM)	0.468 ± 0.072	0.200 ± 0.016
artemisinin (nM)	19.8 ± 1.91	18.1 ± 2.58
chloroquine (nM)	377.8 ± 40.9	341.7 ± 29.1
epoxomicin (nM)	9.26 ± 2.04	8.52 ± 1.95
brefeldin A (μM)	1.33 ± 0.02	1.02 ± 0.61
KDU691 (nM)	34.5 ± 7.87	37.5 ± 3.64
KAF156 (nM)	11.5 ± 0.91	12.8 ± 3.23
cycloheximide (nM)	108.5 ± 9.40	96.5 ± 6.67
actinomycin D (nM)	1.29 ± 0.39	1.12 ± 0.41

As with the functional knockout (MFR3-KO) line, we also evaluated the sensitivity of the MFR3-overexpression line against other drugs with established antimalarial activities and varying mechanisms of action. We found that overexpressing PfMFR3 rendered the parasite significantly more sensitive to atovaquone (*p* = 0.0003), demonstrating the opposite phenotype as the line expressing the truncated form of the protein. On the other hand, the control parasite line that had been transfected with the empty vector was similarly susceptible as the MFR3-overexpression line against the other antimalarials that do not target the mitochondrion.

Using confocal microscopy, we then visualized unfixed, thin smears of different stages of intraerythrocytic Dd2_mfr3over parasites to track the location of the GFP-tagged species of MFR3; smears were also stained with DAPI and MitoTracker Red to visualize the parasite nuclei and mitochondria, respectively. Microscopy-based imaging revealed perinuclear distribution of the GFP signal corresponding to the tagged MFR3 transporter. Strikingly, this GFP signal also appeared to colocalize with the MitoTracker Red signal across different stages of parasite blood-stage development, indicating that this orphan transporter localizes to the parasite mitochondrion (Figure 4d). Interestingly, matching the predicted peptide sequence of MFR3 using position-specific iterated alignment (PSI-BLAST), which allows for the identification of more distantly related proteins, we found that this parasite protein is related to the yeast protein FMP42, an uncharacterized integral membrane protein that localizes to the mitochondrion and vacuole. Our observation that MFR3 is located in the parasite mitochondrion corroborates the link between MFR3 and atovaquone sensitivity given that its drug target is cytochrome bc1. Additionally, it also brings to light the possibility of this subcellular organelle being a site of action of both MMV085203 and GNF-Pf-3600 in *P. falciparum*.

### PfMFR3 Modulates Sensitivity to Compounds that Target the Mitochondrion

**Figure 5:**
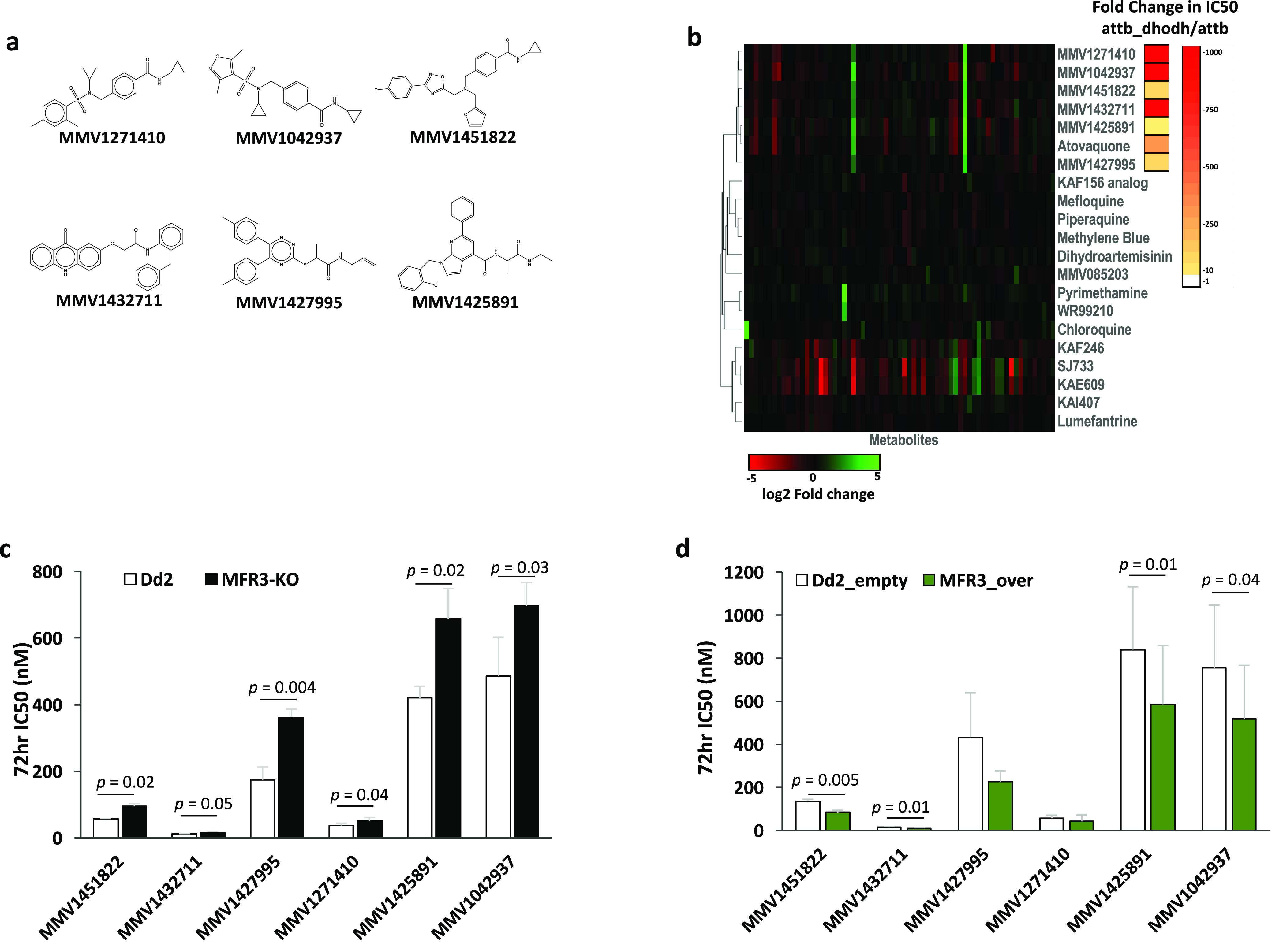
Disruption and overexpression of *pfmfr3* modulates sensitivity against inhibitors of cytochrome bc1. (a) Chemical structures of six structurally diverse compounds (MMV1271410, MMV1042937, MMV1425891, MMV1451822, MMV1432711, and MMV1427995) that target cytochrome bc1 as predicted by metabolomic profiling and the *Sc*DHODH rescue assay. (b) Metabolomic profiles of parasites exposed to the six MMV compounds were coclustered with other clinically relevant antimalarials, including a known inhibitor of cytochrome bc1, atovaquone. Fold-differences in each metabolite can be found in Supplementary Data 1. Each compound was also evaluated for resistance conferred by *Sc*DHODH supplementation, indicating cytochrome bc1 inhibition. Sensitivity of the (c) CRISPR-edited *pfmfr3* mutant expressing the truncated form of the protein (MFR3-KO) as well as the (d) parasite line overexpressing MFR3 was measured against the above-mentioned cyctochrome bc1 inhibitors. Additional data can be found in Table 10. Bars represent the mean ± SD IC_50_ values from at least three independent biological replicates. Pairwise comparisons between parasite lines were performed using the Student’s *t* test.

Given its localization, we also investigated whether the loss of function of this transporter would alter the parasite’s sensitivity against other compounds that, like atovaquone, target the mitochondrion, even without the 1,4-naphthoquinone scaffold. To test this, we chose six compounds (MMV1271410, MMV1042937, MMV1425891, MMV1451822, MMV1432711, and MMV1427995) with antimalarial activity that also inhibit cytochrome bc1 while being structurally dissimilar to atovaquone, MMV085203, and GNF-Pf-3600 (Figure 5a). A comparison of the metabolomic profiles of trophozoite-stage parasites exposed to the six compounds with those of other clinically relevant antimalarial drugs^32,33^ revealed that they all cocluster with atovaquone and demonstrate an increase in levels of *N*-carbamoyl-l-aspartate and dihydroorotate, a metabolic signature that is specifically attributed to inhibitors of cytochrome bc1^32^ (Figure 5b, Supplementary Data 1). Furthermore, we found that all six demonstrated a profound (20- to 3000-fold) rightward shift in IC_50_ in the transgenic *P. falciparum* lines expressing yeast DHODH relative to the attb parent line (Figure 5b), indicating a mechanism of action that involves inhibition of the mitochondrial electron transport chain. Unsurprisingly, the metabolomic profile of MMV085203 did not cocluster with atovaquone or any of the other clinically relevant antimalarials evaluated and shows a completely different pattern of dysregulated metabolites compared to the six other MMV compounds (Figure 5b). This outcome is to be expected given that the DHODH rescue assay has ruled it out as a cytochrome bc1 inhibitor. Interestingly, the metabolic signature for MMV085203 involves a distinct upregulation (>2-fold) of aconitate and fumarate (Supplementary Data 1), both intermediates of the tricarboxylic acid (TCA) cycle, which is a process that takes place in the mitochondrion, further reinforcing the likelihood of MMV085203 engaging a cellular target that is located in this specific organelle.

Evaluating our functional MFR3 knockout line against MMV1271410, MMV1042937, MMV1425891, MMV1451822, MMV1432711, and MMV1427995, we found that disruption of this putative mitochondrial transporter rendered the parasite significantly less sensitive to all six inhibitors of cytochrome bc1 (Figure 5c, Table 10), just as we previously observed with atovaquone. Inversely, overexpression of MFR3 led to diminished 72 h IC_50_ values for all six compounds tested with the increase in sensitivity coming up to significance for MMV1451822, MMV1432711, MMV1425891, and MMV1042937 (Figure 5d, Table 10), and although we have yet to identify the specific function of this novel putative transporter within the parasite, the fact that the disruption and overexpression of this protein leads to a significant alteration in drug sensitivity across a set of structurally diverse molecules that target the mitochondrion reveals the potential of MFR3 to be a relevant multidrug-resistance factor in malaria.

**Table 10:** 72 h IC_50_ Values of Dd2 vs MFR3-KO and Dd2_empty vs MFR_over Strains against Mitochondrial Inhibitors

IC_50_(nM)	Dd2	MFR3-KO	Dd2_empty	MFR3_over
MMV1451822	56.9 ± 0.9	94.57 ± 8.5	134.43 ± 9.9	83.10 ± 10.6
MMV1432711	11.35 ± 1.3	16.50 ± 2.2	13.78 ± 3.9	9.40 ± 3.3
MMV1427995	173.63 ± 39.5	362.17 ± 24.4	432.53 ± 207.2	225.33 ± 51.4
MMV1271410	37.00 ± 7.0	51.10 ± 9.9	56.22 ± 14.4	41.80 ± 29.5
MMV1425891	420.60 ± 34.9	658.30 ± 90.4	840.25 ± 291.2	585.58 ± 272.9
MMV1042937	486.40 ± 116.3	695.53 ± 71.1	755.02 ± 291.1	518.03 ± 248.5

## Discussion

Directed evolution of drug resistance has long been a staple technique for identifying drug targets and mechanisms of resistance in the human malaria parasite.^3,9^ Using this method, we were able to identify and validate a novel mitochondrial transporter in *Plasmodium falciparum*, *pfmfr3* (PF3D7_0312500),^20^ as a mediator of resistance against a number of known mitochondrial inhibitors as well two structurally related compounds with unknown mechanisms of action, MMV085203 and GNF-Pf-3600. While *pfmfr3* is expressed throughout the blood (sexual and asexual), liver, and mosquito stages of the life cycle of *P. falciparum*,^34,35^ genome-wide mutagenesis and knockout screens in *Plasmodium falciparum*(36) and *Plasmodium berghei*(37) demonstrate ready mutability of *pfmfr3*, indicating that it is not essential to the intraerythrocytic development of the parasite. Despite the observation that nonsynonymous mutations in this gene are overrepresented in MMV085203/GNF-Pf-3600-resistant parasite lines derived from two genetically distinct backgrounds, the nonessentiality of this particular gene product, coupled with its function as a transporter, suggests that it is not an actual drug target but rather a shared resistance mechanism.

**Figure 6:**
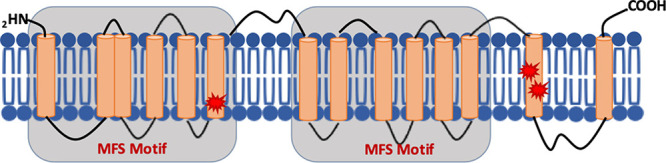
Predicted structure of *S. cerevisiae* ARN1. Protein schematic of yeast ARN1 together with the mutations identified by *in vitro* evolution and whole genome analysis.^38^ Predicted transmembrane domains are marked in orange, and mutations are marked in red stars.

The involvement of MFS transporters in mediating resistance against MMV085203 is also substantiated by directed evolution experiments in a yeast model^38^ where a genetically modified strain of *S. cerevisiae* was subjected to increasing concentrations of the antimalarial drug. Notably, three of the seven clones that demonstrated diminished susceptibility to MMV085203 bore mutations in *ScARN1*, which encodes an iron siderophore transporter, with one of the resistant lines having only a single polymorphism in this gene, while two resistant clones gained mutations in *ScAFT1*, a transcription factor that modulates expression of a cluster of genes, including *ScARN1*,^39^ that are involved in iron homeostasis.^40^ One of four homologous genes in the yeast genome (ARN1–4) that are regulated by AFT1, *ScARN1*, encodes a transport protein that, like PfMFR3, belongs to the MFS superfamily of transporters. Its predicted topology reveals the presence of 14 transmembrane domains with the segment spanning the first 12 transmembrane helices exhibiting homology to other MFS transporters found in bacteria^41,42^ (Figure 6). Depending on extracellular concentration of its siderophore iron substrate, ARN1 is trafficked between the plasma membrane and endosomal compartments in the cytoplasm.^43,44^ Five out of the seven MMV085203-resistant yeast lines acquired mutations that are likely to alter the functionality of this transporter, which points to the involvement of ARN1 in modulating sensitivity to this molecule. Furthermore, outcomes from both yeast and parasite resistance models reinforce the role of these MFS transporters as possibly driving cellular uptake or efflux of MMV085203 and other structurally related molecules.

Denoted as an “orphan” transporter, the mechanism of transport and substrate specificity of MFR3 in *Plasmodium falciparum* is still unknown. The major facilitator superfamily is one of the largest family of transporters and demonstrates immense sequence and functional diversity, and the fact that MFR3 does not strongly resemble any other prokaryotic or eukaryotic protein in the current databases makes it difficult to speculate on its biological function within the parasite. On the other hand, *pbmfr3*, the rodent ortholog of *pfmfr3*, has been shown to be important in sporozoite formation and male gamete exflagellation.^45^ Interestingly, RNA-seq of male and female gametocytes in *P. berghei* revealed that *pbmfr3* is significantly overexpressed in male gametocytes versus female gametocytes,^46^ while in the case of *P. falciparum*, MFR3 is 2-fold more abundant among females versus males.^47^ This differential level of *pfmfr3* expression might also play into the differential susceptibility of *P. falciparum* gametocytes to MMV085203. A prior dual gamete formation screen on the malaria box demonstrated that, while MMV085203 effectively inhibits stage V male gametocytes, it shows no activity against females.^14^ This sex-specific expression pattern could suggest a role for *pfmfr3* in the development of transmission stages in *P. falciparum*. Given its possible function as a mitochondrial transporter, this is not surprising as the parasite mitochondrion is known to undergo significant expansion and activation during gametocytogenesis,^48^ and gametocytes display higher levels of glucose utilization and TCA function,^49^ reflecting increased energy demands for the subsequent stages of the life cycle.

We also determined that, despite the structural similarity across MMV085203, GNF-Pf-3600, and atovaquone, neither MMV085203 nor GNF-Pf-3600 targets the mETC. This result is also supported by the discrepancy in susceptibility of sexual stages of the parasite against these three drugs, wherein atovaquone has been shown to be inactive against late stage *P. falciparum* gametocytes,^50,51^ while both MMV085203 and GNF-Pf-3600 remain efficacious at inhibiting stage V gametocytes.^13^ Additionally, a previous malaria box screen for inhibitors of the parasite enzyme thioredoxin reductase (TrxR) identified MMV085203 as demonstrating the highest level of TrxR inhibition, in contrast with atovaquone, which proved inactive against the enzyme.^52^ An essential component of redox homeostasis maintenance, two isoforms of TrxR, is produced by the parasite from the same *pftrxr* locus with one isoform located in the cytosol and the other localizing to the parasite mitochondrion.^53^ Interestingly, metabolomic profiling of parasites treated with MMV085203 demonstrated a distinct upregulation of aconitate, which is an intermediate formed as citrate is being converted to isocitrate in the tricarboxylic acid cycle. This reaction is catalyzed by aconitate hydratase, or aconitase, an enzyme encoded by PF3D7_1342100 or *pfirp* that has also been found to be located in the mitochondrion.^54^ While not essential for asexual blood-stage growth, PfIRP is important to sexual-stage development, since knocking out this gene renders the parasites unable to form mature gametocytes.^55^ The gametocyte-specific activity of MMV085203, coupled with its effect on aconitate levels in treated parasites, suggests that PfIRP could be another attractive candidate for further investigation as a drug target for MMV085203. Taken together with our results showing localization of MFR3 to the mitochondrion, all of these findings further bolster the possibility of this organelle as one of the sites of action for MMV085203/GNF-Pf-3600.

For certain compounds containing a 1,4-naphthoquinone moiety, their cytotoxic effect is tied to their activity as “subversive substrates” of NADPH-dependent disulfide reductases (such as thioredoxin reductase and glutathione reductase), which leads to inhibition of the physiological reaction catalyzed by these enzymes while also resulting in the production of reactive oxygen species and subsequent disruption of hemoglobin digestion;^56,57^ it is possible that this is one of the mechanisms through which MMV085203 and GNF-Pf-3600 are acting against the parasite.

Our observation that the genetic changes in *pfmfr3* alters the parasite’s drug response against multiple mitochondrial targeting compounds bearing dissimilar chemical scaffolds and likely involving different mechanisms of action demonstrates the potential of MFR3 as a significant mediator of antimalarial multidrug resistance. Essential biological pathways occurring in the mitochondrion such as the mitochondrial electron transport chain^58,59^ and the tricarboxylic acid cycle^49,55^ involve key players that are attractive druggable targets for developing clinical antimalarials. Cytochrome bc1, for example, has been shown to be inhibited by a wide variety of chemotypes^60−63^ and can be targeted for prophylactic, therapeutic, and transmission-blocking purposes.^64^ In addition, the shared resistance caused by disruption of MFR3 against three compounds bearing a 1,4-naphthoquinone scaffold could also speak to a general involvement of this protein in transporting compounds having a similar structure. In addition to MMV085203, GNF-Pf-3600, and atovaquone, the *pfmfr3* Q487E single point mutant also showed 3-fold resistance against another compound with a naphthoquinone group, GNF-Pf-3703 (data not shown). If this is indeed the case, MFR3 activity could be a significant correlate of resistance against a multitude of candidate antimalarials given that naphthoquinone derivatives have immense potential as antiplasmodial leader molecules and have been widely used for the development of many compound series.^56,65−71^ Importantly, polymorphisms in *pfmfr3* have been found to naturally exist in parasite populations in the field with over 50% occurring in transmembrane domains.^72^ Although none of the genetic alterations that were identified in this study have been documented in clinical isolates, one cannot rule out the possibility of these natural mutations leading to modifications in transporter activity and specificity and eventually contributing to clinical drug resistance.

While a number of plasmodium transport proteins have the potential to be attractive therapeutic targets, the fact that PfMFR3 is not essential for parasite growth and replication in the asexual blood stage precludes it from being an ideal antimalarial drug target. Furthermore, it is naturally polymorphic among field isolates, which suggests a higher likelihood of drug resistance emerging against inhibitors of this putative transporter. Overall, these two important considerations impart only a modest clinical impact to the therapeutic inhibition of this protein.

Nevertheless, its role as a transporter in an important and highly druggable organelle make it more interesting as a possible multidrug resistance factor. Further investigation, therefore, into the function of this as-yet-uncharacterized transporter could provide new insight into general parasite biology and lead to a better understanding of what drives resistance in malaria.

## Methods

### *In Vitro* Culture of *P. falciparum*

Two strains (Dd2 and 3D7) of *P. falciparum* were used for *in vitro* drug selection. Continuous cultivation was performed under standard conditions as previously described.^73^ Parasites were grown in human O-positive (O^+^) whole blood obtained from the Blood Bank of The Scripps Research Institute (TSRI) (La Jolla, CA). Leukocyte-free erythrocytes are washed and then stored at 50% hematocrit in RPMI 1640. The evaluation of parasitemia and parasite morphology was performed using a microscopic evaluation of thin blood smears that were first fixed with methanol (Merck) and then stained with Giemsa (Sigma).

### *In Vitro* Selection of Drug-Resistant *P. falciparum*

*In vitro* selection for MMV085203-resistant parasites was performed on a 3D7 background of *P. falciparum*. Parasites were exposed to a progressively increasing drug concentration of MMV085203 starting at 20 nM and eventually culminating at a final exposure concentration of 6× the starting dose (120 nM) after 6 months of selection. In the case of GNF-Pf-3600, *in vitro* selection for resistance was performed on two parental strains, 3D7 and Dd2. Parasites were exposed to stepwise-increasing concentrations of GNF-Pf-3600 starting from 20 and 35 nM for 3D7 and Dd2, respectively. Over the course of 4 months, the drug concentration used for continuous exposure was increased up to 6× the starting dose (125 nM) for 3D7 and 4× the starting dose for Dd2 (150 nM). Once a detectable rightward shift in IC_50_ was detected using the 72 h drug sensitivity assay, MMV085203- and GNF-Pf-3600-resistant clones were obtained through limiting dilution. Clones were then cultivated and phenotyped to confirm resistance, and their genomic DNA was subsequently sent out for whole genome sequencing. Nontreated control parasite lines for 3D7 and Dd2 were maintained in parallel throughout the course of *in vitro* selection.

### 72 h Drug Sensitivity Assay Using SYBR Green I

Drug sensitivities of *Plasmodium falciparum* were evaluated using a SYBR green I-based fluorescence assay.^74^ Briefly, ring-stage parasites growing in a synchronous culture were synchronized by treatment with 5% (w/v) sorbitol. They were then incubated for 72 h in 96-well plates in a 12-dose titration of each drug at a final parasitemia of 0.6% and 2% hematocrit. After 72 h, a 1:1000 mixture of SYBR Green I (Invitrogen) in lysis buffer (0.16% Saponin, 1.6% Triton X-100, 5 mM EDTA, and 20 mM Tris-HCl) was added to each well, and plates were incubated in the dark overnight. Parasite viability was quantified on the basis of a fluorescence readout using a Synergy HTX Multi-Mode Microplate Reader (BioTek). Assays were performed with at least three independent biological replicates with each replicate consisting of technical duplicates. IC_50_’s were then calculated using the drc package in R.

### Whole Genome Sequencing and Variant Calling

To obtain genomic DNA (gDNA) from clonal parasite samples, infected RBCs were washed with 0.05% saponin and gDNA was isolated using a DNeasy Blood and Tissue Kit (Qiagen) according to the standard protocols. Sequencing libraries were prepared with the Nextera XT kit (Cat. No. FC-131-1024, Illumina) via the standard dual index protocol and sequenced on the Illumina HiSeq 2500 in RapidRun mode to generate paired-end reads 100 bp in length. Reads were aligned to the *P. falciparum* 3D7 reference genome (PlasmoDB v13.0) using the previously described Platypus pipeline.^75^ A total of 20 clones were sequenced to an average coverage of 83× with an average of 98.5% of reads mapping to the reference genome. Following alignment, SNVs and INDELs were called using GATK HaplotypeCaller and filtered according to GATK’s best practice recommendations.^76^ Variants were annotated using SnpEff^77^ and further filtered by comparing those from resistant clones to the parent clone, such that only a mutation present in the resistant clone but not the sensitive parent clone would be retained. Since all parasite lines were cloned before sequencing, only variant calls with >90% reads mapped to the alternate allele were considered for resistance conferral. No CNVs were detected in any of the samples following a previously described analysis protocol.^58^

### Disruption of *pfmfr3* Using CRISPR/Cas9

The CRISPR-Cas9 plasmid pDC2-coCas9-U6.2-h*dhfr*^78^ was used to introduce the *pfmfr3* N279 fs mutation (originally detected the MMV085203-resistant clone 3D7-3B3) into *P. falciparum* Dd2 (Figure 2a). The codon for asparagine at residue 279 has a single adenosine (A) deletion resulting in a frameshift and premature termination (N279 fs). The protein size is thus shortened from 579 to 280 amino acid residues. A donor fragment of 577 bp, centered on the frameshift mutation, was synthesized along with silent “shield” mutations at the binding site for the gRNA (TGATAATCAGCTTGTATCAG) located 29 bp upstream of the desired mutation. PCR genotyping of the target locus in cloned transfectants revealed that the entire plasmid recombined into the genome possibly as a result of the double-strand break and homologous-directed repair events by the CRISPR-Cas9 system. However, sequencing of this genomic region confirmed that this recombination event still resulted in a truncated, nonfunctional version of *pfmfr3*. The resulting transgenic parasites were subjected to a 72 h drug sensitivity assay against MMV085203 to confirm the drug resistance phenotype. All primers used in this study are listed in Table 11.

**Table 11:** Primers and Oligonucleotides Used in This Study

name	sequence	description
p282	AACATATGTTAAATATTTATTTCTC	for genotyping of recombinant *pfmfr3* locus
p283	AGGGTTATTGTCTCATGAGCGG	for genotyping of recombinant *pfmfr3* locus
p1277	TGACAGATATCCTGTGGAAGATATCG	for genotyping of recombinant *pfmfr3* locus
p1281	GTGAGGCAAATGTATTTATTATACC	for genotyping of recombinant *pfmfr3* locus
F1_mfr3_avrII	ATCGCCTAGGATGAAAAAAGTAAAGG	for amplification of full length *pfmfr3* cDNA
R1_mfr3_mfeI	ATCGCAATTGTTACATTTGCTGTAG	for amplification of full length *pfmfr3* cDNA
F2_mfr3_mfeI	ATCGCAATTGGATATATATCTTTAGTG	for amplification of full length *pfmfr3* cDNA
R2_mfr3_nheI	ATCGGCTAGCCTTTGAAGAAGGAAGGG	for amplification of full length *pfmfr3* cDNA
BSD_F	ATCAACAGCATCCCCATCTC	for amplification of the BSD resistance cassette
BSD_R	ATGCAGATCGAGAAGCACCT	for amplification of the BSD resistance cassette
mfr3_GFP_F	TCACCTTCACCCTCTCCACT	for amplification of the *pfmfr3*-GFP junction on the overexpression/tagging episome
mfr3_GFP_R	CCAAAGGCAATAGCTCAAGG	for amplification of the *pfmfr3*-GFP junction on the overexpression/tagging episome
rrs_qpcr_F	GAGTACCCCAATCACCTACA	for qPCR (ΔΔCt), reference
rrs_qpcr_R	AAGAGATGCATGTTGGTCATTT	for qPCR (ΔΔCt), reference
mfr3_qpcr_F	CCAAAGGCAATAGCTCAAGG	for qPCR (ΔΔCt), target
mfr3_qpcr_R	TTGAAGAAGGAAGGGAAATCA	for qPCR (ΔΔCt), target

### Generation of *pfmfr3*-GFP Overexpressing Lines

A Dd2 strain was made to episomally overexpress a GFP-tagged version of PfMFR3 using the pDC2-*cam*-*mrfp*-2A–*gfp* plasmid^31^ (Figure 4a). The RFP-2A segment was excised, and the full-length coding sequence of *pfmfr3*, generated from gene-specific PCR amplification of total cDNA extracted from wild-type Dd2, was inserted into the vector upstream of the GFP tag. A parasite line transfected with the empty plasmid was also generated and grown in 2.5 μg/mL blasticidin (BSD) alongside the overexpression lines to serve as a vector control. PCR genotyping confirmed the presence of the empty vector and MFR3-GFP-containing episome in each respective parasite line (Figure 4b).

To confirm overexpression, real-time quantitative PCR (relative quantification) was also performed using gene-specific primers that are able to interrogate both the endogenous and episomal *pfmfr3* transcripts and using PF3D7_1218600 (Arginyl-tRNA synthetase, *pfrrs*) as the reference gene. The parasite line transfected with the empty plasmid was used as the control for ΔΔCt.

Total RNA was extracted from synchronized trophozoite-stage parasite cultures using the TRIZol-Chloroform method.^79^ First strand cDNA synthesis was first carried out on 50 ng of each transfectant line using oligo-dT-primed reverse transcription using SuperScript II Reverse Transcriptase (Invitrogen) according to the manufacturer’s instructions. The resulting first strand product was then used as template for real-time qPCR using Power SYBR Green Master Mix (Applied Biosystems) and using *pfmr3* (target gene) and *pfrrs* (reference gene primers). The ΔΔCt method was used to analyze the relative changes in the expression level of *pfmfr3*, where ΔΔCt = [(Ct of sample *pfmfr3* – Ct of sample *pfrrs*) – (Ct of control *pfmfr3* – Ct of control *pfrrs*)] and 2^–ΔΔCt^ is the fold-difference in gene expression. All primers used in this study are listed in Table 11.

### Imaging of *pfmfr3*-GFP Overexpressing Parasites

Asynchronous blood-stage parasites episomally expressing MFR3-GFP were incubated with 200 nM MitoTracker Red (Invitrogen) for 30 min and subsequently washed three times with warm (37 °C), 1× PBS. Thin blood smears using the blood (2–4 μL) were then generated and mounted with Vectashield with DAPI (Vector Laboratories) and then sealed with glass coverslips. Images were acquired using a Zeiss LSM880 with Airyscan confocal microscope (63× oil immersion lens); diode laser power was set to 2% for 405, 488, and 561 nm. The images were captured and processed using the confocal ZEN software (Black edition, Zeiss).

### Metabolomic Profiling of *P. falciparum* Blood-Stage Parasites

The metabolite profiling of drug-treated, trophozoite-stage parasites was performed as previously described.^32,33^ Briefly, highly synchronized, MACS-purified parasites aged 24–36 h post-invasion were treated with compound at a dose of 10× IC_50_ for 2.5 h alongside an untreated control. Parasitized red blood cells were extracted with 90% methanol containing 0.5 mM ^13^C^15^N-labeled aspartate as an internal standard and stored at −80 °C prior to downstream processing. Samples were subsequently resuspended in HPLC-grade water mixed with 1 mM chlorpropamide as an additional internal standard and analyzed by ultrahigh-performance liquid chromatography mass spectrometry (UHPLC-MS). Hierarchical clustering of metabolite profiles was performed using Cluster 3.0 and visualized using Treeview 3.0.

## Supporting Information Available

Table of parasite metabolites profiled after drug treatment (XLSX)
